# Roles of extracellular polymeric substances in uranium immobilization by anaerobic sludge

**DOI:** 10.1186/s13568-019-0922-2

**Published:** 2019-12-11

**Authors:** Hai-Ling Zhang, Meng-Xi Cheng, Shi-Cheng Li, He-Xiang Huang, Wei-Dong Liu, Xian-Jin Lyu, Jian Chu, Huan-Huan Ding, Dong Zhao, Yong-Peng Wang, Feng-Yu Huang

**Affiliations:** 1 Institute of Materials, China Academy of Engineering Physics, Jiangyou, 621907 Sichuan China; 2 School of Environment and Resources, Southwest University of Science & Technology, Mianyang, 621010 Sichuan China

**Keywords:** Uranium, Extracellular polymeric substances (EPS), Anaerobic sludge, Analysis of variance, Grey relational analysis

## Abstract

The specific roles of extracellular polymeric substances (EPS) and how factors influenced EPS’s roles during U(VI) immobilization are still unclear. In this study, high content of U with the main form of nanoparticles was detected in EPS, accounting for 10–42% of total U(VI) removal. EPS might be utilized as energy source or even as electron donors when external carbon source was unavailable. The influencing degree of each experimental parameter to uranium (U) removal process was elucidated. The influential priority to U(IV)/U(VI) ratios in sludge was as follows: acetate, U(VI), and nitrate. The influential priority to total EPS contents was as follows: U(VI), nitrate and acetate. The complex interaction mechanism between U(VI) and EPS in the U immobilization process was proposed, which might involve three ways including biosorption, bioreduction and bioprecipitation. These results indicate important and various roles of EPS in U(VI) immobilization.

## Introduction

Uranium (U) pollution is an issue of global concern due to its high toxicity and radioactivity. In some U mining regions, the concentration of U in groundwater, mainly in the form of uranyl ion (UO_2_^2+^) or uranyl carbonate complexes [e.g. UO_2_(CO_3_)_2_^2−^], can reach as high as 50 mg/L (Wu et al. 2006; Sarri et al. 2013). Bioremediation offers a cost-effective approach to tackle such U contamination through microbial reduction of soluble U(VI) to insoluble U(IV) (e.g. UO_2_) (Martins et al. 2010; Newsome et al. 2014; Suzuki et al. 2016; Suriya et al. 2017). Recent studies showed that anaerobic granular sludge enriched with U(VI)-reducing microorganisms could effectively and stably reduce U(VI) in specific wastewaters without any external electron donors (Luna-Velasco et al. 2010; Luo et al. 2007; Tapia-Rodriguez et al. 2011), indicating a great prospect for anaerobic granular sludge to control U contamination.

As a major component of anaerobic sludge, EPS form a matrix where microbial cells are enclosed. Our previous work showed a significant accumulation of U in EPS of anaerobic sludge (Zhang et al. 2017). However, the dynamic change of U species and interactions between U and EPS are still unclear. Single particle inductively coupled plasma-mass spectrometry (ICP-MS) offers an efficient tool to identify the U species in solution (Degueldre et al. 2006; Zhang et al. 2017). It can not only distinguish the U in soluble and particulate forms, but also reveal size distribution information of the U particles. Using this technique, more detailed information about the roles of EPS in U removal by anaerobic sludge system could be obtained.

U immobilization by EPS could be influenced by factors like external electron donors or nitrate. In order to maintain the reducing environment and ensure efficient U bioreduction, organic carbon is usually supplied continuously or intermittently. The supply rate of organic carbon could affect not only the U(VI)/U(IV) equilibrium but also the content and composition of EPS, consequently the U-EPS interaction might also be affected (Wan et al. 2008; Tokunaga et al. 2008). Besides, due to the frequent use of nitric acid in uranic extraction process, nitrate usually coexists with U in the groundwater at concentrations up to 2000 mg/L, which might cause biologically catalyzed reoxidation of U(IV) (Luna-Velasco et al. 2010; Moon et al. 2007; Tokunaga et al. 2008). However, the detailed impacts of organic carbon load and nitrate concentration on the U immobilization by EPS in anaerobic sludge are still to be clarified.

This work aims to elucidate the possible roles of EPS in U removal by anaerobic sludge. The migration kinetics and transformation pathways of U among bulk liquid, EPS and sludge in the U immobilization process were analyzed. The effects of factors [influent U(VI) concentration, external electron donors and nitrate] on the U removal by microbes and EPS were explored by analysis of variance (ANOVA) coupled with grey relational analysis (GRA). Specifically, ANOVA was applied to confirm whether the differences between the data of various environmental parameters are statistically significant, while GRA was performed to evaluate the influential priority of various factors (Chen and Syu 2003; Moran et al. 2006; Xu et al. 2011). The present work may benefit a better understanding on the mechanism of U removal from groundwater by anaerobic sludge.

## Materials and methods

### U(VI) immobilization tests by anaerobic sludge

U(VI) immobilization tests were conducted in 1200-mL serum bottles where 600-mL synthetic U contaminated groundwater mixed with anaerobic sludge was injected, leaving 600-mL headspace. Serum bottles were operated in batch modes to explore the effects of U(VI) load, external electron donors and nitrate on immobilization and details of experimental parameters were listed in Table 1. U added in synthetic groundwater was uranyl sulfate (UO_2_SO_4_·3H_2_O, 99.99%, Hubei Chushengwei Chemical Co., Ltd, China) and 10 mM stock solution was prepared in advance. Synthetic groundwater also contain other minerals, whose specific components were shown in Additional file 1: Table S1 (Tapia-Rodriguez et al. 2010). Careful steps were taken to ensure the normal startup of each bottled bio-reactor. First, the minerals solution without U was boiled for 10 min, and sparged with nitrogen to remove dissolved oxygen. Then NaHCO_3_ was dosed to the minerals solution when it was cooled down to room temperature. The final concentration of NaHCO_3_ was 1 g/L. Subsequently, certain U stock solution and anaerobic sludge were successively added into the medium to their respective pre-set concentrations. The volatile suspended solids (VSS) concentration of anaerobic sludge was 1500 mg/L. Before being sealed with butyl rubber stoppers and aluminum tearoff seals, these bottled bio-reactors were sparged with nitrogen again to further remove the residual oxygen. Each batch test continuously ran 7 days with three parallel bottled bio-reactors and their temperatures were controlled at 30 ± 1 °C. During the running time, 11-mL mixed solution was taken at certain intervals. 1-mL solution was used for the sequential extraction of U in sludge, while 10-mL for the EPS extraction.

**Table 1:** Experimental design

Test	U(VI)	Acetate	Nitrate	VSS (g/L)
Control	×	×	×	1.5
Test 1	10 mg/L	×	×	1.5
Test 2	50 mg/L	×	×	1.5
Test 3	50 mg/L	10 mM	×	1.5
Test 4	50 mg/L	10 mM	20 mg/L	1.5

The anaerobic sludge was obtained from a full-scale upflow anaerobic sludge blanket reactor (Hefei, Anhui, China) treating starch wastewater, then stored anaerobically at 4 °C. The VSS content of sludge was 65% and the specific acetoclastic methanogenic activity was 350 mg COD/g VSS/day (COD: chemical oxygen demand). To facilitate subsequent EPS extraction, the sludge granules were firstly washed with de-ioned water, then grinded to flocs before inoculation in our test.

### Fractionation of U in sludge and EPS extraction

**Fig. 1:**
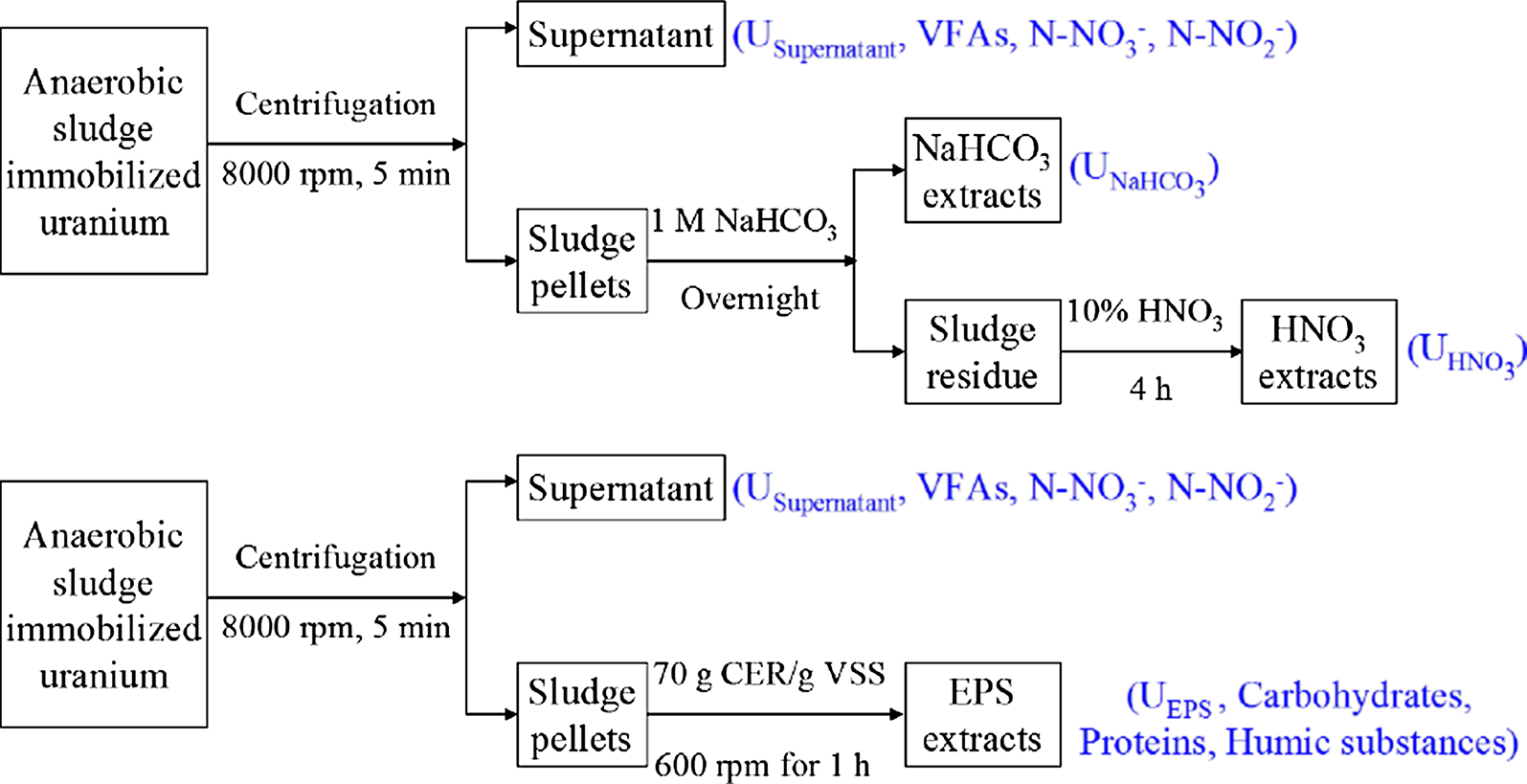
Schematic diagrams for analyses of U, VFAs, N–NO_3_^−^ and N–NO_2_^−^ in the supernatant, and U in NaHCO_3_–HNO_3_ fractionation of sludge; and U, carbohydrates, proteins and humic substances in EPS

Sequential NaHCO_3_ and HNO_3_ extraction approach was applied to fractionate the U in the anaerobic sludge in our test due to its effectiveness (Luna-Velasco et al. 2010; Tapia-Rodriguez et al. 2011), whose schematic diagram is shown in Fig. 1. In the approach, NaHCO_3_ extraction represented adsorbed soluble U(VI) ions and U(VI) precipitates, while HNO_3_ extraction represented insoluble U(IV) [like insoluble UO_2_, or even U(IV)-phosphate]. As shown in Fig. 1, EPS were extracted from the anaerobic sludge using the cation exchange resin (CER) method (more details were shown in Additional file 1). Besides good performance in EPS extraction, CER method has also been proved effective in EPS-associated U extraction in our previous study (Zhang et al. 2017). High extracellular U extraction efficiency and low U(IV) re-dissolution degree was achieved with CER method.

### Chemical analysis

All chemicals used were of analytical grade. The suspended solids (SS) and VSS of the anaerobic granular sludge, the concentrations of N–NO_3_^−^ and N–NO_2_^−^ were measured according to the Standard Methods (APHA 1998). The volatile fatty acids (VFAs) concentration was measured by a two-point titration method (Jenkins et al. 1983; Ripley et al. 1986), and titration pH was selected at 5.75 and 4.3 (Lahav and Morgan 2004). The concentrations of U in supernatant (U_Supernatant_), EPS (U_EPS_), NaHCO_3_ extracts (${\text{U}}_{{\text{NaHCO}}_{3}}$) and HNO_3_ extracts (${\text{U}}_{{\text{HNO}}_{3}}$) were analyzed by ICP-MS (NEXION 350, Perkin Elmer), and all samples were acidified in 5% HNO_3_. The total U in sludge (U_Sludge_) was calculated as the sum of ${\text{U}}_{{\text{NaHCO}}_{3}}$ and ${\text{U}}_{{\text{HNO}}_{3}}$. The calculation results were in good agreement with the results obtained by hot concentrated HNO_3_ extraction (Additional file 1: Table S2). The total content of EPS (TC_EPS_) as well as contents of carbohydrates, proteins and humic substances in EPS extracts were determined as described previously (Frølund et al. 1996). Functional groups present in the EPS were analyzed by Fourier transform infrared spectroscopy (FTIR) and titration methods. The presence of riboflavin and cytochromes C was identified by UV/visible absorption spectroscopy. The two analytical procedures are shown in Additional file 1.

### Analysis of EPS extracts by single particle ICP-MS

The soluble and particulate U fractions in the EPS extracts were quantified by single particle ICP-MS, and so did the size distribution of particulate U. Details of analytical procedure are shown in Additional file 1. Single particle ICP-MS was also applied to analyze the transformation of U in EPS after reoxidation. Before measurement, the EPS solution was placed in a dust-free enclosure with air exposed and temperature controlled at about 10 °C for 4 days.

### Results analysis

As shown in Table 1, environmental parameters were designed to investigate their effects, which were accomplished by comparing results of two tests with only specific parameter variable. One-way ANOVA method was applied to distinguish whether significant difference existed between/among results obtained by only changing the specific parameter. In the method, the significance level was checked by the probability (*P*) value with confidence of 95%. Significant difference existed when *P *< 0.05 and extremely significant difference existed when *P *≤ 0.01, while insignificant difference existed when *P *≥ 0.05. Meanwhile, the influential priority of various environmental parameters was evaluated using GRA method with grey relational grades γ as the quantitative index. Both ANOVA and GRA were performed by Microsoft excel. Details are shown in Additional file 1.

## Results

### Contents and forms of U in the anaerobic sludge

As shown in Table 1, tests without external carbon source and nitrate added (i.e. test 1 and test 2) were conducted to evaluate the effect of U(VI) load, and initial U(VI) concentrations applied in tests were 10 and 50 mg/L, respectively. Besides, the effects of external carbon source and nitrate were investigated based on test 2, test 3 and test 4. In these three tests, the same U load [U(VI), 50 mg/L] was applied, while 10 mM acetate and 20 mg/L nitrate were selectively added or not.

**Fig. 2:**
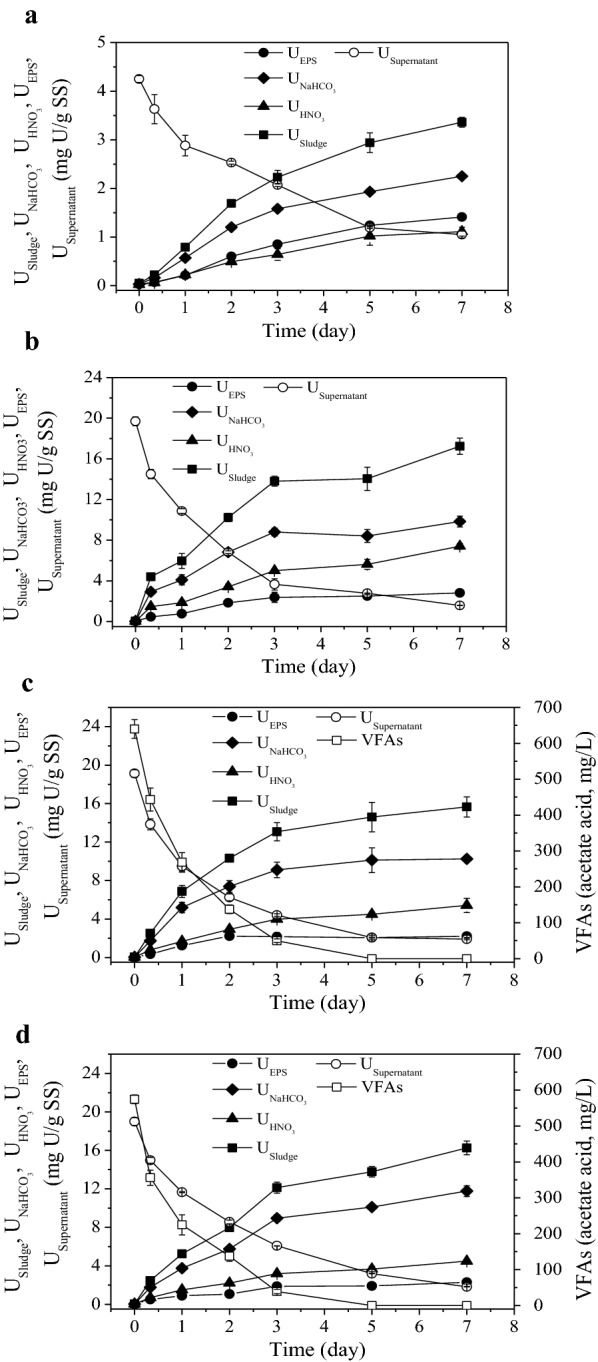
Profiles of: VFAs, U_Supernatant_, U_EPS_, ${\text{U}}_{{\text{NaHCO}}_{3}}$, ${\text{U}}_{{\text{HNO}}_{3}}$ and U_Sludge_ in the time course of U(VI) immobilization by anaerobic sludge. **a** Test 1, 10 mg/L U(VI) without acetate; **b** test 2, 50 mg/L U(VI) without acetate; **c** test 3, 50 mg/L U(VI) with acetate; **d** test 4, 50 mg/L U(VI) with acetate and nitrate

During U(VI) immobilization process by anaerobic sludge in all the tests, VFAs and U (U_Supernatant_, U_EPS_, ${\text{U}}_{{\text{NaHCO}}_{3}}$, ${\text{U}}_{{\text{HNO}}_{3}}$ and U_Sludge_, normalized to SS content of sludge) were analyzed, and results were displayed in Fig. 2. As shown, U_Supernatant_ declined constantly in the process in all tests with U added, which decreased rapidly at the beginning, then decreased more slowly after Day 3. The U removal rates during the initial 3 days were calculated (Additional file 1: Table S3). In addition, U(VI) could be immobilized effectively by anaerobic sludge using endogenous substrate when external carbon source lacked (Fig. 2b), while adding carbon source would apparently accelerate the immobilization process (Fig. 2c).

Different from continuous decline of U_Supernatant_ in the process, U_Sludge_ gradually increased in all the four tests and the trends of U_Sludge_, ${\text{U}}_{{\text{NaHCO}}_{3}}$ and ${\text{U}}_{{\text{HNO}}_{3}}$ were similar (Fig. 2). U in NaHCO_3_ extracts and HNO_3_ extracts represented the adsorbed U(VI) [e.g. U(VI) ions, U(VI)-phosphate minerals] and the reduced U(IV), respectively (Tapia-Rodriguez et al. 2011). U(IV)/U(VI) ratios were calculated, and variations of which in anaerobic sludge during the process are shown in Fig. 4a. Interestingly, the U(IV)/U(VI) ratio of original anaerobic sludge was as high as 0.75, which might be due to the gradual and long anaerobic reduction of tiny natural U existed in the starch wastewater. In tests with U(VI) added, U(IV)/U(VI) ratios decreased significantly after reaction, then gradually increased as time went on.

Results of the U(IV)/U(VI) ratios of anaerobic sludge in tests were analyzed using one-way ANOVA method (Table 2). As shown, only changing U(VI) load (from 10 to 50 mg/L) or adding external carbon source could cause significant difference in U(IV)/U(VI) ratios since both *P* values were equal to 0.024, which also implied that the reduction activity of microorganisms to U(VI) was significantly excited when facing U(VI) of higher influent concentration. As to the effect of adding nitrate or not, it was insignificant based on the data of U(IV)/U(VI) ratios in the whole 7 days (*P *> 0.05), while significant based on the data Day 3 to Day 7 (*P *= 0.028). After Day 3, the role of U(IV) reoxidation by nitrate might escalate (Luna-Velasco et al. 2010; Moon et al. 2007; Tokunaga et al. 2008). The less obvious influence of nitrate on U(IV)/U(VI) ratios was also reflected by GRA result of test 4 (Table 3, Additional file 1: Table S10). As shown, the influential priority to U(IV)/U(VI) ratios was as follows: acetate presence (γ = 0.8618), U(VI) concentrations (γ = 0.8178), and nitrate presence (γ = 0.6707). GRA result of test 3 showed the same influential priority sequence for acetate presence (γ = 0.6066) and U(VI) concentrations (γ = 0.5149).

**Table 2:** ANOVA analysis for ratio of U(IV)/U(VI) in anaerobic sludge

Two group data		Factor	F statistic	*P*
Test 1	Test 2	U(VI) concentration	7.02^a^	0.024^a^
Test 2	Test 3	Acetate presence	7.08^a^	0.024^a^
Test 3	Test 4	Nitrate presence	3.01^a^	0.113^a^
			0.010^b^	0.925^b^
			11.25^c^	0.028^c^

^a^Data from Day 0.3 to Day 7

^b^Data from Day 0.3 to Day 2

^c^Data from Day 3 to Day 7

**Table 3:** GRA analysis for the ratio of U(IV)/U(VI) and U_EPS_/TC_EPS_

Test	Output variable	Grey relational grades of input variable		
		U(VI) concentration	Acetate presence	Nitrate presence
Test 3	U(IV)/U(VI)	0.5149	0.6066	
	U_EPS_/TC_EPS_	0.7670	0.4521	
Test 4	U(IV)/U(VI)	0.8178	0.8618	0.6707
	U_EPS_/TC_EPS_	0.8576	0.4691	0.4649

### Characterization of EPS

**Fig. 3:**
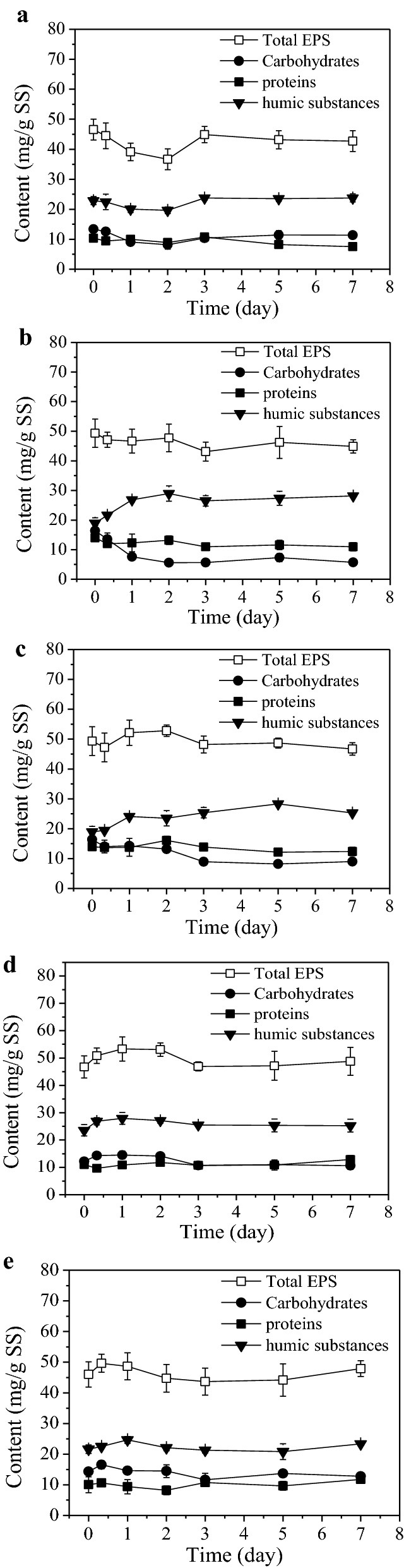
EPS concentrations and compositions in the time course of U(VI) immobilization by anaerobic sludge. **a** The control test; **b** test 1, 10 mg/L U(VI) without acetate; **c** test 2, 50 mg/L U(VI) without acetate; **d** test 3, 50 mg/L U(VI) with acetate; **e** test 4, 50 mg/L U(VI) with acetate and nitrate

Figure 3 shows EPS variations in all the U(VI) immobilization tests by anaerobic sludge, including their contents and main components (carbohydrates, proteins and humic substances). As shown, different EPS trends displayed under different experimental conditions. In the control test, EPS content declined from 46.6 to 36.7 mg/g SS in the initial 2 days (Fig. 3a), then gradually increased up to 42.7 mg/g SS during the later period. The trends of three main components were similar to that of EPS.

GRA and ANOVA methods were also applied to analyze the influential priority of parameters to total EPS content and results were as follows: U(VI) concentrations, nitrate presence and acetate presence (GRA analysis results, Additional file 1: Table S13; ANOVA analysis results, Additional file 1: Table S4). Results of two methods agreed well. According to ANOVA analysis, the effect of 10 mg/L U(VI) on EPS content was insignificant based on results from control test and test 1 (*P *= 0.325 > 0.05), while results from test 1 and test 2 indicated that the EPS contents in two tests were significantly different with the raising of influent U(VI) concentration from 10 to 50 mg/L (*P *= 0.024). Insignificant difference was shown between EPS contents with or without 10 mM acetate dosed (*P *= 0.056 > 0.05). Nitrate of 20 mg/L was also not enough to cause significant influence to EPS contents (*P *= 0.105 > 0.05).

Notably, extremely significant difference existed in carbohydrates contents when 10 mg/L U(VI) was added or not (*P *< 0.01, Additional file 1: Table S5). In contrast with the control test, obvious decrease in carbohydrates was observed in both test 1 and test 2. The carbohydrates in EPS obviously increased in the initial 2 days when acetate was present, attributing to the absorption of VFAs (Fig. 2c). Content of carbohydrates in EPS in test 3 (acetate: 10 mM) were significantly different (*P *< 0.01, Additional file 1: Table S5) comparing with test 2 (acetate: 0 mM). However, results from test 3 and test 4 illustrated that 20 mg/L nitrate did not bring significant variation to the carbohydrates in EPS (*P *> 0.05). Interestingly, humic substances gradually increased in the process. Humic substances were generated with EPS as part of them, nevertheless, they could be hardly consumed due to their little bioavailability.

### Contents and forms of U in the EPS

**Fig. 4:**
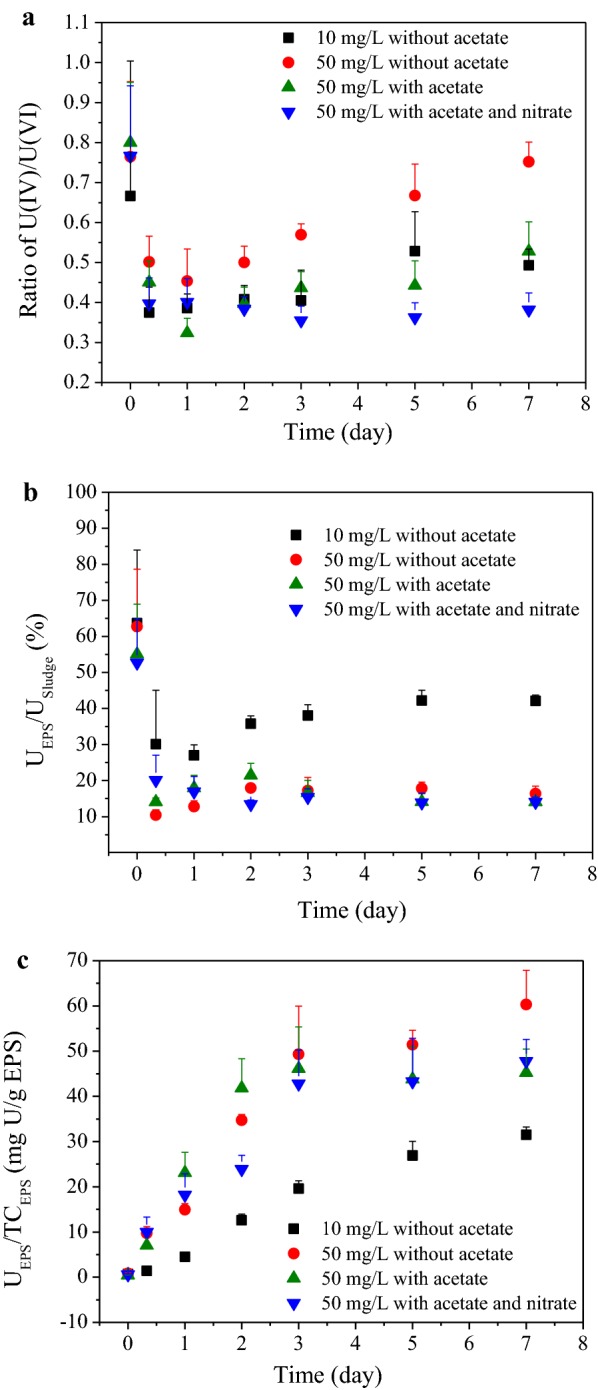
Variations of: **a** ratio of U(IV)/U(VI); **b** U_EPS_/U_Sludge_ and **c** U_EPS_/TC_EPS_ in the time course of U(VI) immobilization by anaerobic sludge (test 1, 10 mg/L U(VI) without acetate; test 2, 50 mg/L U(VI) without acetate; test 3, 50 mg/L U(VI) with acetate; test 4, 50 mg/L U(VI) with acetate and nitrate)

As shown in Fig. 2, trends of U_EPS_ during the U(VI) immobilization process was similar in tests with 50 mg/L U(VI) (test 2, test 3 and test 4, respectively shown in Fig. 2b–d), which gradually increased in the initial 3 days and then nearly stabilized with much lower increasing rates. Unlike tests with 50 mg/L U(VI), U_EPS_ increased almost linearly during the whole process in the test with 10 mg/L U(VI) (test 1, shown in Fig. 2a). Ratios of U_EPS_/U_Sludge_ were calculated and results are shown in Fig. 4b. As shown, there seemed to be a negative correlation between U_EPS_/U_Sludge_ ratios and influent U(VI) concentrations. The original anaerobic sludge owned the highest U_EPS_/U_Sludge_ ratio of 58.5%, and U_EPS_/U_Sludge_ ratios in tests with 10 mg/L U(VI) (27.0–42.0%) was significantly higher (*P *= 0.000, Additional file 1: Table S8) than that in tests with 50 mg/L U(VI) (10.4–21.5%). The dynamic change of U content in EPS (expressed as U_EPS_/TC_EPS_) during the U(VI) immobilization process is shown in Fig. 4c. The variation trends of U_EPS_/TC_EPS_ were similar to that of U_EPS_ (Fig. 2).

**Fig. 5:**
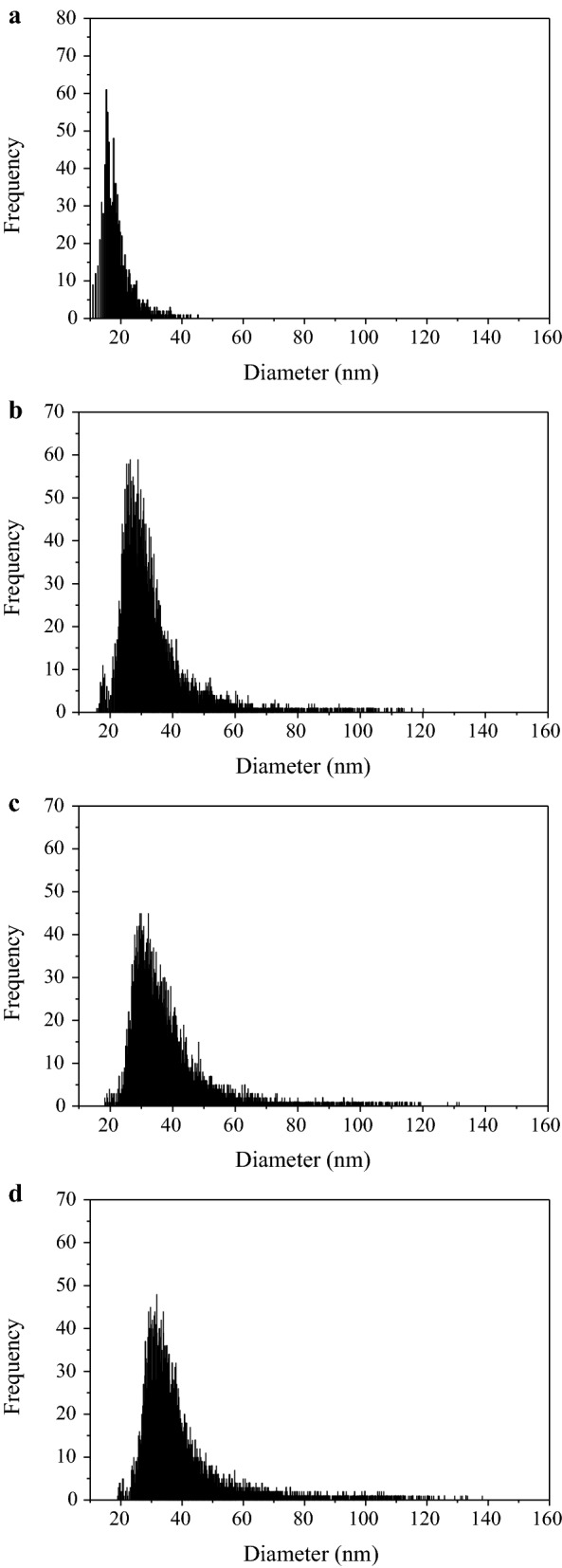
Size distribution of nano-sized U particles in EPS extracts (diluted 200 times, each frequency equals to particle concentration of about 21.2 parts per mL). **a** Original sludge, Day 0; **b** Day 3; **c** Day 5; **d** Day 7. Influent concentration of U(VI) was 50 mg/L and no acetate was added

The forms of U in EPS extracts were measured using ICP-MS (Fig. 5, Additional file 1: Figure S4). Fractions of soluble and particulate U in EPS, and also size distribution of particulate U are shown in Table 4. Over 80% of U in the EPS was mainly present in the form of nano-sized particulates under all conditions (Fig. 5d, Additional file 1: Figure S4a, b, c). Take EPS from the test 2 [U(VI): 50 mg/L; no acetate and nitrate added] as example, U particles inside gradually formed and grew larger during the initial 7 days, with concentrations increasing from 58,646 to 384,747 parts/mL and mean size from 19.9 to 43.5 nm (Fig. 5 and Table 4). The mean size of U particulates was all around 42 nm in U-loaded tests at Day 7. EPS were also characterized using the FTIR spectrometry (Additional file 1: Figure S5). Abundant functional groups (hydroxyl, carboxylic group, etc.) were detected. Meanwhile, high density of bicarbonate and phosphate ions were found in EPS using UV/visible spectra (Additional file 1: Table S14). Riboflavin was also found in EPS (Additional file 1: Figure S6).

**Table 4:** Fractions of soluble and particulate U and size distribution of particulate U in EPS extracts detected by ICP-MS (diluted 200 times)

Test	Time	Soluble (%)	Particulate (%)	Mean size (nm)	Particle concentration (parts/mL)
Test 1	Day 7	15.1 ± 1.3	84.9 ± 5.3	42.8 ± 3.5	90,667 ± 4431
Test 2	Day 0	9.66 ± 0.7	90.3 ± 3.3	19.9 ± 0.9	58,646 ± 1947
	Day 3	13.1 ± 1.0	86.9 ± 4.6	37.6 ± 2.4	364,762 ± 6707
	Day 5	15.5 ± 0.3	84.5 ± 6.1	41.7 ± 4.2	374,427 ± 7619
	Day 7	13.8 ± 0.7	86.2 ± 5.2	43.5 ± 1.1	384,747 ± 3377
Test 3	Day 7	14.5 ± 0.6	85.5 ± 3.3	42.3 ± 2.7	146,833 ± 7786
	Day 7 after 4-day air exposure	6.4 ± 0.4	93.6 ± 6.7	36.2 ± 1.3	523,062 ± 1044
Test 4	Day 7	14.7 ± 0.8	85.3 ± 2.9	43.6 ± 3.5	155,788 ± 3328

The transformation of U in EPS after reoxidation was also analyzed (Additional file 1: Figure S4c, d and Table 4). Interestingly, the mean size of U particles in EPS from test 3 [U(VI): 50 mg/L; acetate: 10 mM; nitrate: 0 mg/L] decreased from 42.3 to 36.2 nm, while the particle concentration significantly increased from 146,833 to 523,062 parts/mL after air exposure of 4 days.

## Discussion

### U immobilization by the anaerobic sludge

The obvious difference among U removal rates during the initial 3 days indicated that U(VI) removal kinetics could be influenced by environmental parameters, such as U(VI) load, external carbon source or co-existing contaminants (like nitrate). Compared with other tests, the U(VI) removal rate in test 1 was rather low, which might be ascribed to insufficient gap between the influent U(VI) concentration (10 mg/L) and the attainable U(VI) concentration after anaerobic sludge treatment. Reduction of U(VI) to U(IV) precipitation inevitably contributed to U immobilization, while nitrate could meanwhile cause substrate consumption through denitrification (Additional file 1: Figure S2) and U(IV) reoxidation (Luna-Velasco et al. 2010; Moon et al. 2007; Tokunaga et al. 2008), which both had adverse effects to U(VI) immobilization. Hence, the U(VI) immobilization rate in test 4 was lower than that in test 3 when 20 mg/L nitrate was added (Additional file 1: Table S3, Fig. 2d).

As reported, the U(VI)/U(IV) equilibrium would shift with the increasing of carbonate concentration caused by microbial respiration, and the addition of external carbon source would lead to higher fraction of adsorbed U(VI) (Wan et al. 2008; Tokunaga et al. 2008). Similar results were obtained in our test, and further than that, the influential priority of external parameters to the U(VI)/U(IV) ratios were calculated using both ANOVA method and GRA method. Results of two methods were consistent, with priority as follows: acetate presence, U(VI) concentrations and nitrate presence.

Taken results of U(IV)/U(VI) ratios, U_Supernatant_ and U_Sludge_ together, it could be concluded that most U(VI) was combined to sludge through adsorption or complexation before reduction to U(IV), and combination of U(VI) and sludge was much faster than U(VI) bioreduction by sludge. Notably, no matter what effects caused by external carbon source or nitrate, advantageous or disadvantageous, anaerobic sludge showed high efficiency in U(VI) removal in our tests, which was all higher than 90% when influent U(VI) concentration was 50 mg/L (Fig. 2b–d).

### Variations of EPS contents and compositions

Different EPS trends under different experimental conditions implied that all parameters including the influent U(VI) concentration, external electron donors and nitrate should have different influences on EPS production and consumption processes. It was known that EPS production and consumption were simultaneous and quite complex, and apparent EPS content was the result of equilibrium of both processes. EPS could be produced by processes like cell lysis or degradation of external carbon source, while consumed when microorganisms was in adverse situations (like endogenous respiration or decay phase) to support microbial survival.

Microorganisms were under endogenous respiration conditions in the control test with U, acetate and nitrate absent. As stated above, EPS would be degraded to support microbial survival in such adverse situation, leading to a significant decline of EPS content in the initial 2 days (Fig. 3a). However, cell lysis gradually increased as the adverse situation continued (Additional file 1: Figure S3) and became the predominant process affecting the apparent content of EPS, resulting gradually increased EPS content during the later period.

According to GRA analysis results (Additional file 1: Table S13) and ANOVA analysis results (Additional file 1: Table S4), the influential priority to total EPS contents was as follows: U(VI) concentrations, nitrate presence and acetate presence, and the effects of the latter two were insignificant. As reported, exposure to toxic substances higher than certain concentrations would stimulate obvious cell lysis, EPS excretion, and also inhibition of microbial respiration (Nwachukwu and Pulford 2011; Wang et al. 2015; Sheng et al. 2010). Indeed, influent U(VI) of 50 mg/L rather than 10 mg/L triggered statistically significant changes in EPS contents based on comparing results of control test, test 1 and test 2 (Additional file 1: Table S4, *P* was equal to 0.024 and 0.325, respectively). Supply of external carbon source avoided using EPS as nutrition, and also alleviated cell lysis which generated EPS, resulting in insignificant difference between EPS contents with or without 10 mM acetate dosed (*P *= 0.056 > 0.05). Although denitrification, which was accompanied with addition of nitrate (Additional file 1: Figure S2), would consume acetate and further influence EPS content, nitrate of 20 mg/L was also not enough to cause significant influence (*P *= 0.105 > 0.05).

Comparing with the control test, the carbohydrates in EPS obviously decreased in test 1 and test 2, demonstrating that the consumption of carbohydrates would be stimulated after adding U(VI) in situations lacking external carbon source. Carbohydrates were consumed not only to provide nutrition for microorganisms, but probably also to serve as electron donors for U(VI) bioreduction. Compared with test 2 (acetate: 0 mM) and test 3 (acetate: 10 mM), content of carbohydrates in EPS were significantly different (*P *< 0.01, Additional file 1: Table S5), obvious increase of carbohydrates was observed in EPS in the initial 2 days when acetate was present, attributing to the absorption of VFAs (Fig. 2c). However, results from test 3 and test 4 illustrated that 20 mg/L nitrate did not bring significant variation to the carbohydrates in EPS (*P *> 0.05).

### U immobilization by the EPS

The U_EPS_/U_Sludge_ ratios, representing the contribution of EPS to U(VI) removal by anaerobic sludge, ranged from 10.4 to 42.0% when 10–50 mg/L U(VI) was artificially added (Fig. 4), indicating that the roles of EPS in U(VI) removal could not be neglected. Besides, external carbon source and nitrate had nearly little effects on U_EPS_/U_Sludge_ ratios (Table 3, Additional file 1: Table S8).

The dynamic change of U content in EPS (U_EPS_/TC_EPS_) was characterized, which revealed similar variation trends to that of U_EPS_ (Fig. 2), indicating that U was gradually immobilized by EPS and it had little relationship with the production and hydrolysis of EPS (Fig. 3). As known, the adsorption equilibrium of heavy metal ions by EPS can be achieved in a short time. Hence, gradual increase of U_EPS_/TC_EPS_ implied that other U immobilization mechanisms should be involved in addition to U(VI) adsorption. Besides, comparing with acetate and nitrate, 50 mg/L U(VI) exhibited much more significant effect on the ratio of U_EPS_/TC_EPS_ (Table 3, Additional file 1: Table S9). More U could be reserved in per unit mass of EPS under conditions of high influent U(VI) concentration and no external carbon source.

The soluble and particulate U in EPS was distinguished and characterized using ICP-MS (Fig. 5, Additional file 1: Figure S4). Over 80% of U in the EPS was present as nano-sized particulates and mean size at Day 7 in all U-loaded tests was around 42 nm, indicating that the size of U particulate in EPS was hardly affected by environmental parameters. Based on characterization results of EPS by FTIR and UV/visible methods, functional groups (hydroxyl, carboxylic group, etc.) possessed by EPS and ions (like bicarbonate ions and phosphate ions) existing in EPS were all favorable components for EPS to immobilize U. Functional groups could favor U immobilization through biosorption or chelation, while ions might reacted with uranyl ions, forming U(VI)-carbonate precipitates or U(VI)-phosphate precipitates and leading to occurrence of mineralization (Newsome et al. 2014). Bioreduction of U(VI) to U(IV) by EPS could also exist, as reported in previous works, including ours (Cao et al. 2011; Zhang et al. 2017). Riboflavin (Additional file 1: Figure S6) and humic substances (Fig. 3) contained in EPS might contribute to their U(VI) reducing capacity (Xiao et al. 2017; Xiao and Zhao 2017). Hence, soluble U(VI), U(VI) precipitates and U(IV) particles were all accumulated in EPS of anaerobic sludge.

The transformation of U in EPS after reoxidation was also analyzed (Additional file 1: Figure S4c, d and Table 4). The generation of large amounts of smaller U(VI) precipitates should be result of the reaction between local phosphate or carbonate ions inside EPS and soluble U(VI) ions gradually released from the reoxidation process of U(IV) precipitate, since EPS could act as a template for mineral nucleation (Lin et al. 2012). Reoxidation of U(IV) was much slower than reaction between phosphate or carbonate and U(VI) ions because the later was a homogeneous reaction in EPS solution. Slow release of one reactant would promote the formation of precipitates with small particle size based on the theory of crystallization kinetics. As a note, reoxidation of U(IV) inside the EPS should be even slower, since the conditions of EPS after extraction from anaerobic sludge were totally different from their original condition in anaerobic sludge. The original EPS were more like bio-colloids rather than solution. The original EPS could prevent the remobilization of U(VI) ions to some extent especially when the anaerobic sludge was under micro-aerobic condition.

### Possible roles of EPS in the U immobilization process

**Fig. 6:**
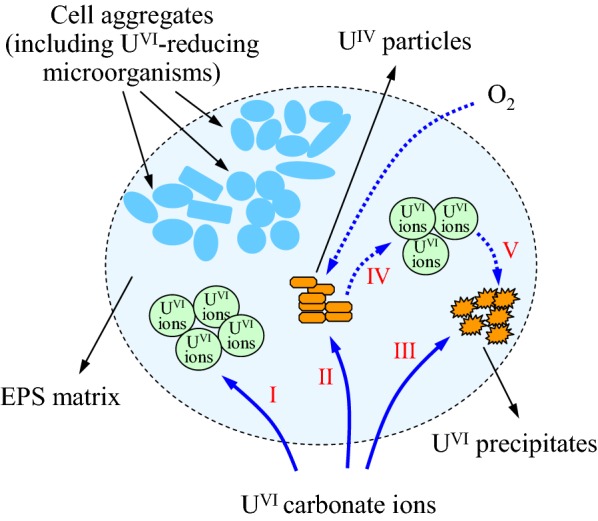
The supposed roles of EPS in the U(VI) immobilization process by anaerobic sludge. Process I: U(VI) ions were adsorbed; Process II: U(VI) ions were reduced to U(IV) particles; Process III: U(VI) ions were precipitated to U(VI) particles; Process IV, V: if oxygen entered, U(IV) particles might dissolve to U(VI) ions and form U(VI) precipitates

Above results show that U immobilization by EPS was complex and at least three ways should be involved (Fig. 6), including biosorption of soluble U [e.g. UO_2_^2+^, UO_2_(CO_3_)_2_^2−^ etc., process I], bioreduction of soluble U(VI) to U(IV) precipitates (process II), and bioprecipitation to form U(VI) particles such as U(VI)-phosphate precipitates (reaction product of adsorbed phosphate and UO_2_^2+^, process III). Notably, in addition to UO_2_ particles, U(IV)-phosphate precipitates might also be contained in the U(IV) precipitates (Newsome et al. 2015). Besides, when oxygen entered, U(IV) particles dissolved to soluble U(VI) ions (process IV) and then formed U(VI) precipitates (process V).

In absence of external carbon source, the EPS (mainly the carbohydrates) might be degraded as energy source for the maintenance of microorganisms, or even as electron donors for U(VI) bioreduction. Besides, EPS could contribute more to the U(VI) removal in lower U(VI)-level systems. Therefore, U immobilization by EPS of anaerobic sludge in short-term operations could be affected by factors like influent U(VI) concentration and providing external carbon source or not. Whatever effects, it was proved that EPS would play important roles in U(VI) immobilization, especially in systems with low U(VI)-level and without external carbon source. It needs to be noted that all tests in this work were run for a short period of 7 days. The anaerobic sludge has been proved long-term (373 days) effective on U(VI) removal without any external electron donors, and reoxidation of U(IV) in the presence of nitrate (about 30 mg/L) became obvious as time went on (Tapia-Rodriguez et al. 2011). Then, the roles of EPS in U(VI) immobilization by anaerobic sludge in long-term operations need further investigations.

High content of nano-sized U particles could be reserved in EPS during U(VI) immobilization process by anaerobic sludge. EPS’s contribution to total U(VI) immobilization process accounted for 10–42% as experimental parameters varied. With ANOVA and GRA methods, the influencing degree of each experimental parameter to U removal process was elucidated. The complex interaction mechanism between U(VI) and EPS in the U immobilization process was proposed, which might involve three ways including biosorption, bioreduction and bioprecipitation. All results imply that EPS have played a significant role in the process of U(VI) immobilization by anaerobic sludge and deserve more attention.

## Supplementary information

**Additional file 1: Table S1.** The components of the minerals solution. **Table S2.** The fractionation of U in the sludge. **EPS extraction by CER method.** **Table S3.** U(VI) removal rates in the initial three days. **Statistical significance test. Grey relational analysis.** **Fig. S2.** N-NO_3_^−^ and N-NO_2_^−^ concentrations during U(VI) immobilization by anaerobic sludge. Influent concentrations of U(VI), acetate and nitrate were respectively 50 mg/L, 10 mM and 20 mg/L. **Fig. S3.** Nucleic acids concentration during U(VI) immobilization by anaerobic sludge without U(VI), acetate and nitrate (control test). **Quantification of the mineral fraction of nano-sized U in the EPS. FTIR analysis of the EPS. Measurement of bicarbonate and phosphate of the EPS.** **Table S14.** Bicarbonate and phosphate concentration in the EPS extracts from the original anaerobic sludge. **UV/visible absorption spectroscopy of the EPS**.
