# Supplementary material for: Roles of extracellular polymeric substances in uranium immobilization by anaerobic sludge
Source: AMB Express. 2019 Dec 11;9:199. doi: 10.1186/s13568-019-0922-2 (PMC6906280; doi:10.1186/s13568-019-0922-2)
Supplement: Supplementary file 1 — Additional file 1: Table S1. The components of the minerals solution. Table S2. The fractionation of U in the sludge. EPS extraction by CER method. Table S3. U(VI) removal rates in the initial three days. Statistical significance test. Grey relational analysis. Fig. S2. N-NO3− and N-NO2− concentrations during U(VI) immobilization by anaerobic sludge. Influent concentrations of U(VI), acetate and nitrate were respectively 50 mg/L, 10 mM and 20 mg/L. Fig. S3. Nucleic acids concentration during U(VI) immobilization by anaerobic sludge without U(VI), acetate and nitrate (control test). Quantification of the mineral fraction of nano-sized U in the EPS. FTIR analysis of the EPS. Measurement of bicarbonate and phosphate of the EPS. Table S14. Bicarbonate and phosphate concentration in the EPS extracts from the original anaerobic sludge. UV/visible absorption spectroscopy of the EPS. [file 13568_2019_922_MOESM1_ESM.doc]

**Additional Information**

**Roles of extracellular polymeric substances in uranium immobilization by anaerobic sludge**

Hai-Ling Zhang^1,#^, Meng-Xi Cheng^1,#^, Shi-Cheng Li^1^, He-Xiang Huang^1^, Wei-Dong Liu^1^, Xian-Jin Lyu^1^, Jian Chu^1^, Huan-Huan Ding^1^, Dong Zhao^1^ Yong-Peng Wang^1,*^, Feng-Yu Huang^2 *^

^1^Institute of Materials, China Academy of Engineering Physics, Jiangyou, Sichuan, 621907, China ^2^School of Environment and Resources, Southwest University of Science & Technology, Mianyang, Sichuan, 621010, China

* **Corresponding author**:

Yong-Peng Wang

E-mail: [747506327@qq.com](mailto:747506327@qq.com ); Telephone and fax: +86 816 3626951

Feng-Yu Huang

E-mail: [huangfengyu1993@126.com](mailto:huangfengyu1993@126.com); Telephone and fax: +86 816 3626356

**^#^ The two authors contributed equally to this work.**

**Table S1. The components of the minerals solution**

| Bulk elements | Concentration (mg/L) |
| --- | --- |
| NH_4_HCO_3_ | 5 |
| K_2_HPO_4_ | 2 |
| MgCl_2_ | 2.1 |
| Ca(OH)_2_ | 1 |
| yeast extract | 0.33 |
|  |  |
| Trace elements | Concentration (μg/L) |
| H_3_BO_3_ | 0.5 |
| FeSO_4_·7H_2_O | 28 |
| ZnSO_4_·7H_2_O | 1.1 |
| CuSO_4_·5H_2_O | 1.6 |
| MnSO_4_·H_2_O | 2.5 |
| (NH_4_)_6_Mo_7_O_24_·4H_2_O | 2.0 |
| KAl(SO4)2·12H_2_O | 1.75 |
| CoSO_4_·7H_2_O | 23.6 |
| NiSO_4_·6H_2_O | 1.13 |
| Na_2_SeO_3_·5H_2_O | 1 |
| Na_2_WO_4_·2H_2_O | 5.2 |
| EDTA | 10 |

**Table S2. The fractionation of U in the sludge**

| Sludge sample | NaHCO_3_ extracts | HNO_3_ extracts | Hot concentrated HNO_3_ extracts | Recovery  (NaHCO_3_-HNO_3_ extraction) |
| --- | --- | --- | --- | --- |
|  | mg U/g SS | mg U/g SS | mg U/g SS | % |
| 1 | 6.52 | 0.60 | 7.20 | 98.9 |
| 2 | 4.16 | 0.31 | 4.51 | 99.1 |
| 3 | 8.74 | 1.12 | 10.1 | 97.6 |

**EPS extraction by CER method**

The EPS extraction process was carried out in an anaerobic glove box (99.9% N_2_) at room temperature. Before extraction, a certain amount of sludge solution taken at each given interval was centrifuged at 8000 rpm for 5 min. Then the sludge pellets were washed twice with 100 mM anaerobic NaCl solution (previously boiled and sparged with N_2_) to remove any residual soluble substances in media. After that, the pellets were resuspended with 100 mM anaerobic NaCl solution to the original volume. The sludge mixture was stirred afterwards for 1 h at 600 rpm in a 50-mL beaker with an appropriate amount of resin (DOWEX MARATHON C, Na^+^-form, 20-50 mesh, Sigma-Aldrich Inc., USA) (Frølund et al. 1996). Thereafter, the suspensions were centrifuged at 10000 rpm for 10 min and subsequently at 14510 rpm (equivalent to 20000 g) for 20 min, and the supernatant was collected without further treatment (D’Abzac et al. 2010; Bourven et al. 2011), withholding the EPS-associated U precipitates.

**
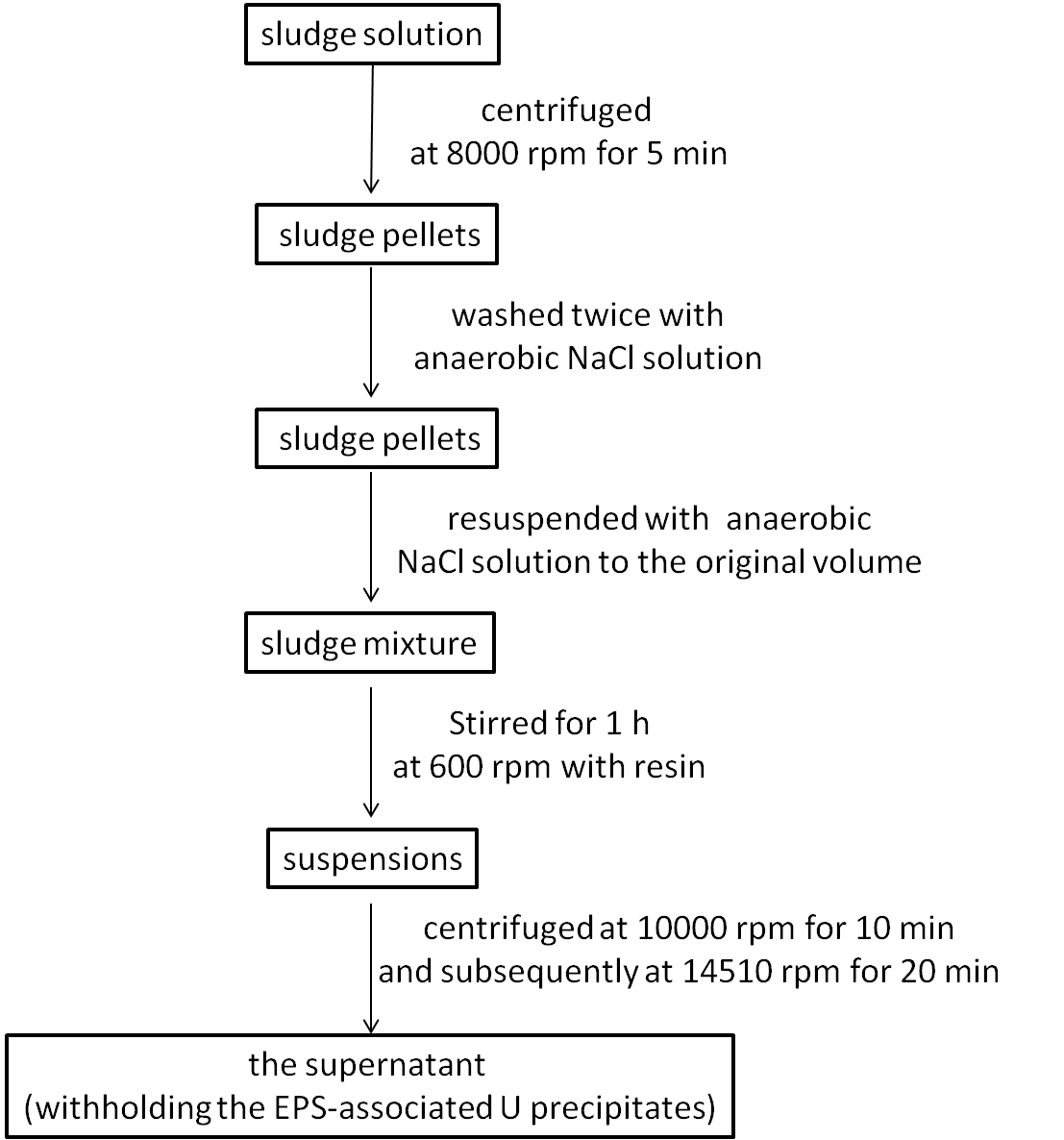
**

**Fig. S1. EPS extraction procedure by CER method**

**References**

Bourven I, Joussein E, Guibaud G (2011) Characterisation of the mineral fraction in extracellular polymeric substances (EPS) from activated sludges extracted by eight different methods. Bior. Technol. 102, 7124-7130.

Frølund B, Palmgren R, Keiding K, Nielsen PH (1996) Extraction of extracellular polymers from activated sludge using a cation exchange resin. Water Res. 30, 1749-1758.

D’Abzac P, Bordas F, Joussein E, van Hullebusch E, Lens PNL, Guibaud G (2010) Characterization of the mineral fraction associated to extracellular polymeric substances (EPS) in anaerobic granular sludges. Environ. Sci. Technol. 44, 412-418.

**Table S3. U(VI) removal rates in the initial three days**

| Test | Main parameters in each test | U(VI) removal rates  (mg U/(g SS·d)) | R^2^ |
| --- | --- | --- | --- |
| 1 | 10 mg/L U without acetate | 0.67 | 0.91 |
| 2 | 50 mg/L U without acetate | 4.9 | 0.93 |
| 3 | 50 mg/L U with acetate | 6.0 | 0.90 |
| 4 | 50 mg/L U with acetate and nitrate | 4.0 | 0.93 |

Those U_Supernatant_ values in the initial three days of each test almost decreased linearly. The U(VI) removal rates were calculated according to the slope of linear fitting equation for each test.

Statistical significance test

One-way ANOVA was usually applied to analyze whether obvious difference existed between/among experimental results under only one variable. For this work, F-test was performed by Microsoft Office Excel to analyze the statistical significance between data of two groups with the confidence of 95%. The data included the total content of EPS (TC_EPS_), carbohydrates, proteins and humic substances in EPS, ratio of U(IV)/U(VI), U_EPS_/TC_EPS_ and U_EPS_/U_Sludge_ in U(VI) immobilization process by anaerobic sludge. P-value (*P*) represents the significance level, which means extremely significant when *P*≤0.01, significant when 0.01≤*P*≤0.05 and insignificant when *P*≥0.05. As the same anaerobic sludge was used in all the experiments, all the data at Day 0 in experiments was not checked.

**Table S4. ANOVA analysis for TC_EPS_**

| Data of two groups | | Factor | F statistic | *P* |
| --- | --- | --- | --- | --- |
| Control | Test 1 (10 mg/L U without acetate) | Influent U(VI) concentration | 1.07 | 0.325 |
| Test 1 (10 mg/L U without acetate) | Test 2 (50 mg/L U without acetate) |  | 7.10 | 0.024 |
| Test 2 (50 mg/L U without acetate) | Test 3 (50 mg/L U with acetate) | External electron donors | 4.67 | 0.056 |
| Test 3 (50 mg/L U with acetate) | Test 4 (50 mg/L U with acetate and nitrate) | Nitrate | 3.17 | 0.105 |

**Table S5. ANOVA analysis for carbohydrates in EPS**

| Two group data | | Factor | F statistic | *P* |
| --- | --- | --- | --- | --- |
| Control | Test 1 (10 mg/L U without acetate) | Influent U(VI) concentration | 12.66 | 0.005 |
| Test 1 (10 mg/L U without acetate) | Test 2 (50 mg/L U without acetate) |  | 4.80^a^  10.92^b^ | 0.053^a^  0.011^b^ |
| Test 2 (50 mg/L U without acetate) | Test 3 (50 mg/L U with acetate) | External electron donors | 12.59 | 0.005 |
| Test 3 (50 mg/L U with acetate) | Test 4 (50 mg/L U with acetate and nitrate) | Nitrate | 0.48 | 0.506 |

^a^: data from Day 0.3 to Day 7

^b^: data from Day 1 to Day 7

**Table S6. ANOVA analysis for proteins in EPS**

| Two group data | | Factor | F statistic | *P* |
| --- | --- | --- | --- | --- |
| Control | Test 1 (10 mg/L U without acetate) | Influent U(VI) concentration | 0.61 | 0.452 |
| Test 1 (10 mg/L U without acetate) | Test 2 (50 mg/L U without acetate) |  | 7.58 | 0.020 |
| Test 2 (50 mg/L U without acetate) | Test 3 (50 mg/L U with acetate) | External electron donors | 0.39 | 0.546 |
| Test 3 (50 mg/L U with acetate) | Test 4 (50 mg/L U with acetate and nitrate) | Nitrate | 0.02 | 0.892 |

**Table S7. ANOVA analysis for humic substances in EPS**

| Two group data | | Factor | F statistic | *P* |
| --- | --- | --- | --- | --- |
| Control | Test 1 (10 mg/L U without acetate) | Influent U(VI) concentration | 43.81 | 0.000 |
| Test 1 (10 mg/L U without acetate) | Test 2 (50 mg/L U without acetate) |  | 1.96 | 0.192 |
| Test 2 (50 mg/L U without acetate) | Test 3 (50 mg/L U with acetate) | External electron donors | 6.63 | 0.028 |
| Test 3 (50 mg/L U with acetate) | Test 4 (50 mg/L U with acetate and nitrate) | Nitrate | 5.96 | 0.035 |

**Table S8. ANOVA analysis for ratio of U_EPS_/U_Sludge_**

| Two group data | | Factor | F statistic | *P* |
| --- | --- | --- | --- | --- |
| Test 1 (10 mg/L U without acetate) | Test 2 (50 mg/L U without acetate) | Influent U(VI) concentration | 51.30 | 0.000 |
| Test 2 (50 mg/L U without acetate) | Test 3 (50 mg/L U with acetate) | External electron donors | 0.300 | 0.596 |
| Test 3 (50 mg/L U with acetate) | Test 4 (50 mg/L U with acetate and nitrate) | Nitrate | 0.211 | 0.656 |

**Table S9. ANOVA analysis for ratio of U_EPS_/TC_EPS_**

| Two group data | | Factor | F statistic | *P* |
| --- | --- | --- | --- | --- |
| Test 1 (10 mg/L U without acetate) | Test 2 (50 mg/L U without acetate) | Influent U(VI) concentration | 4.45^a^  6.15^b^ | 0.061^a^  0.038^b^ |
| Test 2 (50 mg/L U without acetate) | Test 3 (50 mg/L U with acetate) | External electron donors | 0.043^a^ | 0.840^a^ |
| Test 3 (50 mg/L U with acetate) | Test 4 (50 mg/L U with acetate and nitrate) | Nitrate | 0.147^a^ | 0.710^a^ |

^a^: data from Day 0.3 to Day 7

^b^: data from Day 1 to Day 7

Grey relational analysis

The grey relational analysis (GRA) method was applied to determine the influential degree of the factors. The calculation method of GRA was described by Xu et al. (2011), and the value of ξ was set as 0.5 in this study (Chen and Syu, 2003).

The analyzed data (output variables) include the ratio of U(IV)/U(VI), U_EPS_/TC_EPS_, TC_EPS_, contents of EPS components (carbohydrates, proteins and humic substances) in four tests of U(VI) immobilization process by anaerobic sludge. Since the influencing factors (independent input variables) should be composed of at least two, the ratio of U(IV)/U(VI) and U_EPS_/TC_EPS_ were only evaluated in the test under 50 mg/L U(VI) with acetate, and the test under 50 mg/L U(VI) with acetate and nitrate. Notably, causal relationship should exist between the output variable and the independent input variable. Therefore, the data of influencing factors need to be transformed. The influencing factors, actual data and the representation were shown in Table S10-S12.

**Table S10. The set of influencing factors for the ratio of U(IV)/U(VI)**

|  | Actual data | Representation |
| --- | --- | --- |
| U(VI) concentration | the ratio of U(IV)/U(VI) in the test under 50 mg/L U(VI) without acetate | single loading stress of U(VI) |
| Acetate concentration | decreasing extent of acetate concentration | increase in adsorbed U(VI) caused by microbial exogenous respiration |
| Nitrate concentration | nitrate concentration | U(IV) reoxidation by nitrate |

**Table S11. The set of influencing factors for TC_EPS_, carbohydrates, proteins and humic substances in EPS**

|  | Actual data | Representation |
| --- | --- | --- |
| Endogenous respiration | corresponding content in the control test | self-consumption of EPS and EPS production through cell lysis |
| U(VI) concentration | decreasing extent of U(VI) concentration | cell lysis under different U(VI) stress |
| Acetate concentration | decreasing extent of acetate concentration | EPS production under exogenous respiration |
| Nitrate concentration | decreasing extent of nitrate concentration | decrease in the extent of EPS production under exogenous respiration |

**Table S12. The set of influencing factors for the ratio of U_EPS_/TC_EPS_**

|  | Actual data | Representation |
| --- | --- | --- |
| U(VI) concentration | the ratio of U_EPS_/TC_EPS_ in the test under 50 mg/L U(VI) without acetate | single loading stress of U(VI) |
| Acetate concentration | decreasing extent of acetate concentration | increase in TC_EPS_ |
| Nitrate concentration | decreasing extent of nitrate concentration | decrease in TC_EPS_ |

**Table S13. GRA analysis for the total content of EPS (TC_EPS_), carbohydrates, proteins and humic substances in EPS**

| Test | Output variable | Grey relational grades of input variable | | | | |
| --- | --- | --- | --- | --- | --- | --- |
|  |  | Endogenous respiration | U(VI) concentration | Acetate concentration | Nitrate concentration |  |
| Test 1  (10 mg/L U without acetate) | TC_EPS_ | 0.7341 | 0.7213 |  |  |  |
|  | Carbohydrates | 0.6917 | 0.5963 |  |  |  |
|  | Proteins | 0.7408 | 0.7213 |  |  |  |
|  | Humic substances | 0.6325 | 0.6778 |  |  |  |
| Test 2  (50 mg/L U without acetate) | TC_EPS_ | 0.5770 | 0.7435 |  |  |  |
|  | Carbohydrates | 0.5771 | 0.6432 |  |  |  |
|  | Proteins | 0.6416 | 0.6748 |  |  |  |
|  | Humic substances | 0.6494 | 0.6057 |  |  |  |
| Test 3  (50 mg/L U with acetate) | TC_EPS_ | 0.6285 | 0.7748 | 0.7628 |  |  |
|  | Carbohydrates | 0.5674 | 0.7600 | 0.8061 |  |  |
|  | Proteins | 0.5606 | 0.6935 | 0.6665 |  |  |
|  | Humic substances | 0.5449 | 0.8486 | 0.7779 |  |  |
| Test 4  (50 mg/L U with acetate and nitrate) | TC_EPS_ | 0.5127 | 0.6256 | 0.5273 | 0.5725 |  |
|  | Carbohydrates | 0.5376 | 0.7308 | 0.7179 | 0.5942 |  |
|  | Proteins | 0.6749 | 0.7155 | 0.6689 | 0.6744 |  |
|  | Humic substances | 0.6074 | 0.6091 | 0.6379 | 0.6329 |  |

**References**

Chen MY, Syu MJ (2003) Film analysis of activated sludge microbial discs by the Taguchi method and grey relational analysis. Bioprocess Biosyst. Eng. 26, 83-92.

Xu J, Sheng G, Luo H, Fang F, Li W, Zeng RJ, Tong Z, Yu H (2011) Evaluating the influence of process parameters on soluble microbial products formation using response surface methodology coupled with grey relational analysis. Water Res. 45, 674-680.

**Fig. S2.** N-NO_3_^-^ and N-NO_2_^-^ concentrations during U(VI) immobilization by anaerobic sludge. Influent concentrations of U(VI), acetate and nitrate were respectively 50 mg/L, 10 mM and 20 mg/L.

**Fig. S3.** Nucleic acids concentration during U(VI) immobilization by anaerobic sludge without U(VI), acetate and nitrate (control test)

**Quantification of the mineral fraction of nano-sized U in the EPS**

Single-particle ICP-MS (NEXION 350, Perkin Elmer) was used to quantify the mineral fraction of nano-sized U in the EPS extracts after 0.22-μm filtration. In its single particle mode, the signal of soluble uranyl ions was obviously different from that of U contained particles. The EPS solution was divided into two equal parts. One part was used to determine the total U concentration in EPS (acidified in 5% HNO_3_), and the other part was used to determine the concentration of soluble U ions. The mineral fraction of EPS-associated U was evaluated by the difference between the concentrations of total U and soluble U ions in EPS.

To characterize size distribution of U particles, a series of silver nanoparticles (Citrate NanoXactTM Silver, nanoComposix Inc.) with known diameter and particle concentration were used as the standard particles. One colloidal particle would be ionized in the plasma torch and form a flash of ions, then display as a transient signal, which could be detected by MS. The signal intensity represents the particle size and the flash frequency represents the particle concentration.

**a**

**b**

**c**

**d**

**Fig. S4.** Size distribution of nano-sized U particles in EPS extracts at Day 7 (each frequency equals to particle concentration of about 21.2 parts per mL). a: diluted 1400 times, test 1; b: diluted 200 times, test 2; c: diluted 200 times, after 4-day air exposure, test 3; d: diluted 200 times, test 4

**FTIR analysis of** **the EPS**

The EPS extracts from the original anaerobic sludge was freeze-dried and analyzed by FTIR (FTIR-650, TianJin GangDong Sci.&Tech. Development Co. LTD, China). As shown in Fig. S3, the peak at 3430 cm^-1^ were the stretching vibration of —OH in the EPS. The absorption bands at 1628 cm^-1^ and 1560 cm^-1^ were associated with the characteristic vibrations of the —CONH— group of Amide I and Amide II in proteins. The band around 1400 cm^-1^ was attributed to stretching vibration of C=O from carboxylic group and deformation vibration of —OH from alcohols and phenol groups. The peak at 1120 cm^-1^ was attributed to stretching vibration of C—O—C group in the carbohydrate cyclic structures.

**Fig. S5.** FTIR spectrum of EPS from the original anaerobic sludge

**Measurement of bicarbonate and phosphate** **of the EPS**

Titration method was used to measure bicarbonate concentration in the EPS extracted from the original anaerobic sludge. Both two end-point (pH=5.0, 4.4) titration (Rieger and Weiland, 2006) and three end-point (pH=5.0, 4.3, 4.0) titration (Kapp, 1984) were applied. PO_4_^3-^ concentration in EPS extracts was measured by ammonium molybdate colorimetry according to the Standard Methods (APHA, 1998).

**Table S14. Bicarbonate and phosphate concentration in the EPS extracts from the original anaerobic sludge**

|  | Concentration  mM/ g SS | | |
| --- | --- | --- | --- |
|  | Two end-point titration | Three end-point titration | Ammonium molybdate colorimetry |
| Bicarbonate | 0.081±0.011 | 0.103±0.021 |  |
| Phosphate |  |  | 0.025±0.001 |

**References**

APHA (1998) Standard methods for the examination of water and wastewater, 20thed., American Public Health Association: Washington, DC.

Kapp H (1984) *Schlammfaulung mit hohem Feststoffgehalt. Stuttgarter Berichte zur Siedlungswasserwirtscaft, Band 86,* Oldenbourg Verlag, Munchen, 300 pp.

Rieger C, Weiland P (2006) *Prozessstrungen frühzeitig erkennen*, Biogas Journal. 4, 18-20.

**UV/visible absorption spectroscopy of the EPS**

UV/visible absorption spectroscopy (UV-1800 spectrometer, Shimadzu Co., Japan) was used to identify the presence of riboflavin or cytochromes C in the EPS extracts of the anaerobic sludge, which were collected at the beginning and the 7^th^ day of the test culturing with 50 mg/L U(VI) and 10 mM acetate. Results of UV/visible absorption spectroscopy of EPS under both conditions, NaCl (100 mM) and pure riboflavin (5 mg/L) were shown in Fig. S4. As shown, riboflavin with the characteristic peak at about 265 nm was detected in EPS from sludge which had reacted with U for 7 days, while not detected in EPS from initial non-reacted sludge. The characteristic peak of riboflavin in our study was consistent with that reported by Xiao (Xiao et al. 2017). However, cytochrome C was not detected in both EPS samples.

**Fig. S6.** UV/visible spectra of 100 mM NaCl, 5 mg/L pure riboflavin and EPS from the anaerobic sludge at the beginning and the 7^th^ day of test culturing with 50 mg/L U(VI) and 10 mM acetate.

**References**

Xiao Y, Zhang E, Zhang J, Dai Y, Yang Z, Christensen HEM, Ulstrup J, Zhao F (2017) Extracellular polymeric substances are transient media for microbial extracellular electron transfer. Sci. Adv. 3, e1700623.
